# Role of Sphingosine Kinase in Type 2 Diabetes Mellitus

**DOI:** 10.3389/fendo.2020.627076

**Published:** 2021-02-09

**Authors:** Yanfei Qi, Wei Wang, Ziyu Song, Gulibositan Aji, Xin Tracy Liu, Pu Xia

**Affiliations:** ^1^ Lipid Cell Biology Laboratory, Centenary Institute of Cancer Medicine and Cell Biology, University of Sydney, Sydney, NSW, Australia; ^2^ Department of Endocrinology and Metabolism, Fudan Institute for Metabolic Diseases, Zhongshan Hospital, Fudan University, Shanghai, China

**Keywords:** insulin resistance, β-cell, sphingolipid, sphingosine 1-phosphate, ceramide

## Abstract

Sphingolipids are a class of essential lipids, functioning as both cell membrane constituents and signaling messengers. In the sphingolipid metabolic network, ceramides serve as the central hub that is hydrolyzed to sphingosine, followed by phosphorylation to sphingosine 1-phosphate (S1P) by sphingosine kinase (SphK). SphK is regarded as a “switch” of the sphingolipid rheostat, as it catalyzes the conversion of ceramide/sphingosine to S1P, which often exhibit opposing biological roles in the cell. Besides, SphK is an important signaling enzyme that has been implicated in the regulation of a wide variety of biological functions. In recent years, an increasing body of evidence has suggested a critical role of SphK in type 2 diabetes mellitus (T2D), although a certain level of controversy remains. Herein, we review recent findings related to SphK in the field of T2D research with a focus on peripheral insulin resistance and pancreatic β-cell failure. It is expected that a comprehensive understanding of the role of SphK and the associated sphingolipids in T2D will help to identify druggable targets for future anti-diabetes therapy.

## Introduction

Type 2 diabetes mellitus (T2D) is a progressive metabolic disease caused by impaired responses to insulin in the target tissues, such as the liver, skeletal muscle and adipose tissue, and inadequate insulin production from pancreatic islets. T2D predisposes to a significantly higher risk of severe health problems in the cardiovascular system, kidney, eyes, and nervous system, leading to reduced life quality and even death. According to the International Diabetes Federation (IDF) statistics in 2019, 1 in 11 adults aged between 20 and 79 are living with T2D, incurring a heavy socioeconomic burden (1). As the largest non-contagious pandemic of the 21^st^ century, T2D is spreading rapidly towards less developed communities and the younger population (1). Despite intensive research for decades, the molecular mechanism underlying the pathogenesis and progression of T2D remains to be identified.

Dyslipidemia has long been recognized as a chief causative factor tying T2D with lifestyle (2). However, with the advancement of understanding in lipids, it is abundantly clear that circulating lipids only represent a small portion of pathogenic factors for T2D. Intraorganic/intracellular lipids contribute to T2D at all stages, from the early prediabetes to the establishment of complications (3, 4). Indeed, T2D is often associated with intracellular lipid dysregulation, as commonly seen in obesity, non-alcoholic fatty liver disease, and lipodystrophy (5, 6). Sphingolipids are a class of essential intracellular lipids that function as both cell membrane constituents and signaling molecules. Sphingosine kinase (SphK), including the two isoforms SphK1 and SphK2, is a key enzyme of the sphingolipid metabolic pathway (7). In recent years, an increasing body of evidence has suggested an important role of SphK in T2D, although a certain level of controversy remains. This review intends to provide a comprehensive review on SphK in the research field of T2D with a focus on peripheral insulin resistance and pancreatic β-cell failure.

## Sphingolipid Metabolic Network

**Figure 1:**
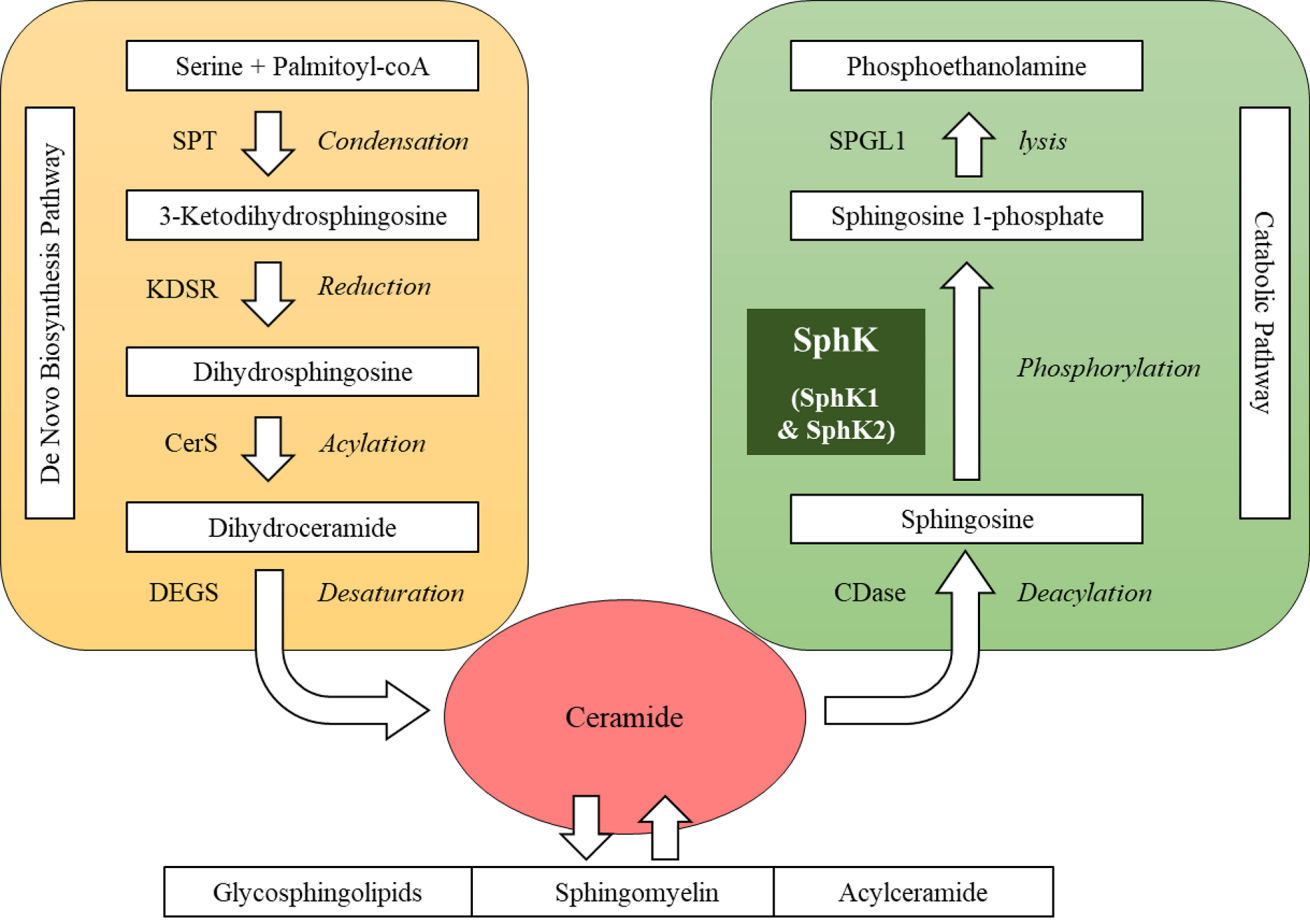
Sphingolipid metabolic network. SPT, serine palmitoyltransferase; KDSR, 3-keto-dihydrosphingosine reductase; CerS, ceramide synthase; DEGS, dihydroceramide desaturase; CDase, ceramidase; SphK, sphingosine kinase; SPGL1, sphingosine 1-phosphate lyase.

Sphingolipids present one of eight major lipid categories. LIPID MAPS, the largest lipid-only database, lists 1,664 curated sphingolipid species (8). Structurally, these sphingolipid metabolites possess a common sphingoid backbone, mainly in 18-carbon length (9, 10). Metabolically, sphingolipid products are interconnected as a network with ceramide as the central hub (**Figure 1**). In the sphingolipid biosynthetic pathway, the first committed step is the formation of sphingoid base from amino acid and fatty acyl-CoA *via* condensation and reduction (9, 11). In the following steps, ceramide is produced on the basis of the sphingoid backbone *via* acylation and desaturation (12, 13). Ceramide can be reversibly converted into complex sphingolipids, such as sphingomyelin, glycosphingolipids and acylceramide (14–16). In the sphingolipid catabolic pathway, ceramide can be hydrolyzed into sphingosine that is subsequently phosphorylated into sphingosine 1-phosphate (S1P) by SphK (17–19). S1P can be irreversibly lysed into non-lipid products to exit the sphingolipid metabolic network (20). SphK is often regarded as a key enzyme in this network primarily for two reasons: a) SphK constitutes the checkpoint that determines the cellular content of “bioactive” sphingolipid products, including ceramide, sphingosine and S1P (18, 21); b) SphK acts as a “rheostat” of sphingolipids, because ceramide and sphingosine often exhibit cellular functions opposing S1P (22). For example, ceramide and sphingosine induce apoptosis, whereas S1P protects cells from death in various types of cells (22).

## Structure of Sphingosine Kinase

There are two mammalian SphK isoforms, designated as SphK1 and SphK2, which are encoded by two different genes located on distinct chromosomes. SphK1 and SphK2 are evolutionarily highly conservative (18, 19). Both of them possess five conserved regions, C1-C5 (18, 19). The C1-C3 and C5 domains in SphK share a high level of sequence homology with phosphofructokinase (PFK)-like superfamily members, such as NAD kinase and diacylglycerol kinase (23). However, the crystalized structure of SphK1 reveals that its lipid-binding pocket is distinctive from NAD kinase and diacylglycerol kinase, indicative of the substrate specificity (24). Whilst the structure of SphK2 has not been characterized yet, sequence alignment analyses reveal that compared with SphK1, SphK2 possesses an additional N-terminal extension and a large insertion at its C-terminal lipid-binding domain (24, 25). SphK2, but not SphK1, possesses a nuclear localization sequence and a nuclear export sequence, which are located in its N-terminal extension and C-terminal insertion, respectively (26). Thus, SphK2 can shuttle between the nucleus and cytoplasm, whereas SphK1 is solely a cytosolic protein. SphK2 is also found to function in the endoplasmic reticulum (ER) and mitochondria (27–29), which might be related to its putative transmembrane domain and BH3 domain (30, 31).

## Biological Functions of Sphingosine Kinase

Both SphK1 and SphK2 are ubiquitously expressed in tissues and blood. SphK1 contributes more to the level of S1P in circulation, while SphK2 is the predominant SphK isoform in the liver, kidney, pancreatic islets and brain (32–34). The ablation of either *Sphk1* or *Sphk2* does not cause any infertility or lethality in mice, whereas knockout of both enzymes results in embryonic death, indicating they are redundant in some essential physiological functions (35). At the cellular level, the primary biological actions of SphK1 are to promote cell proliferation, migration and survival and to inhibit apoptosis, and thus upregulation of SphK1 is often associated with cancer progression, metastasis and a poor prognosis (13, 36, 37). In addition, SphK1 modulates immune response and inflammation (38, 39). In general, the biological function of SphK2 has not been well characterized. In some cases, SphK2 and SphK1 play similar roles because of their overlapping enzymatic activity in the conversion of ceramide and sphingosine into S1P; whereas in others, SphK2 exhibits different or even opposite effects due to distinct tissue distribution and subcellular localization. In particular, the role of SphK2 is highly controversial in terms of pro-apoptotic (27, 31, 40) versus anti-apoptotic (41–43). The mechanism underlying this discrepancy warrants further elucidation.

## Action Modes of Sphingosine Kinase

SphK1 and SphK2 exert their biological functions mainly *via* the following three approaches:

**a) S1P Receptor-Dependent Mode**: SphK often functions *via* its enzymatic product S1P. Once generated by SphK, S1P can be exported out of the cell where it binds to a group of five distinct, specific G protein-coupled receptors (S1P1-5) (44, 45). S1P can also travel to its target tissues *via* its carriers, such as albumin and ApoM-containing high-density lipoproteins (46, 47). Circulating S1P is mainly sourced from the SphK1 in erythrocytes (48). However, a recent study has found that SphK2 also determines the circulating level of S1P in the liver, where it dictates S1P clearance *via* a route of dephosphorylation, re-phosphorylation and lysis (49). Circulating S1P is undoubtedly implicated in whole-body metabolic regulation, but its physiological effects remain elusive. Plasma S1P is found increased in humans and rodents with obesity and insulin resistance (50), whereas another study identifies an opposite trend that serum S1P is decreased along with the development of human insulin resistance and T2D (51). However, the ApoM-bound S1P is recently found metabolically protective against insulin resistance (52). More extensive studies are required to clarify the role of circulating S1P. Cells from different tissues can express one to five different kinds of S1P receptors (53). Upon activation by S1P, S1P receptors trigger cell signaling coordinately or differentially. For instance, both S1P1 and S1P4 are required for T cell migration (54), while both S1P1 and S1P2 convey S1P-mediated protection from excitotoxicity in hippocampal neurons (55). In contrast, S1P1 and S1P2 exhibit opposite effects on pain sensitivity and endothelial dysfunction (56, 57). In addition, S1P receptor signaling is often crosstalk with other receptor-dependent pathways, such as growth factors and cytokines (58–62), leading to a complexity of the actions of S1P.
**b) Intracellular Partner Mode:** SphK and S1P can directly bind to intracellular partners, independent of S1P receptors. For instance, at the plasma membrane, SphK1 and S1P interact with the RING domain of tumor necrosis factor receptor-associated factor 2 (TRAF2), priming the ubiquitination of receptor-interacting serine/threonine-protein kinase 1 (RIP1) and subsequent activation of nuclear factor-κB (NFκB) (63, 64). In the nucleus, SphK2 and its product S1P physically interact with histone deacetylases HDAC1 and HDAC2, implicating in the epigenetic regulation (26). At the inner membrane of mitochondria, SphK2 and S1P bind to prohibitin 2, which is essential for respiratory complex IV assembly and mitochondrial fitness (29). More intraorganellar partners of SphK are to be identified, e.g., in the ER where SphK1 regulates unfolded protein responses (27, 65), endosomes where SphK1 modulates endocytotic recycling (66, 67) and lysosomes where SphK2 mediates autophagy (68).
**c) Substrate Depletion Mode:** Reducing levels of ceramide and sphingosine is often regarded as a mean of S1P-independent function of SphK. For examples, SphK1 counteracts ceramide-induced apoptosis, constituting the basis of cancer drug resistance (69). Overexpression of SphK1 prevents ceramide accumulation in myocytes ameliorates muscle insulin resistance in diet-induced obese mice (70). SphK has also been found to regulate certain cellular functions *via* sphingosine, independent of ceramide or S1P: sphingosine specifically impairs SphK1-mediated endocytotic membrane trafficking (66); sphingosine induces cell cycle arrest in SphK1/SphK2 double knockout murine embryonic stem cells (71); sphingosine accumulation suppresses insulin signaling in SphK2 deficient hepatocytes (7); and sphingosine impairs insulin-mediated glucose uptake in L6 myotubes exposed to palmitate (72).

## Sphingosine Kinase and Hepatic Insulin Resistance

The liver is the central organ in maintaining glucose homeostasis. Hepatic insulin resistance is considered a key pathogenic onset of prediabetes (73). Aberrant lipid metabolism in the liver impairs hepatocyte’s response to insulin, leading to increased glucose production and decreased glucose disposal (73). Conventionally, lipid dysregulation that results in hepatic insulin resistance mainly refers to ectopic fat deposition, as seen in steatotic livers. In accord, 70-80% of T2D subjects have non-alcoholic fatty liver diseases (NAFLD), and the incidence rate of NAFLD in obese, diabetic individuals is more than 90% (5, 74). In the past decade, the role of signaling lipids, including SphK-related sphingolipids like ceramides, sphingosine, and S1P, is emerging in the pathogenesis of hepatic insulin resistance (3, 7, 75).

The role of SphK1 in hepatic fitness is discrepant, mainly because it simultaneously possesses both prosurvival and proinflammatory properties. As a prosurvival factor, SphK1 protects hepatocytes from a variety of stress stimuli. SphK1 protects NAFLD livers from ischemia/reperfusion injury by reducing reactive oxygen species and alleviating oxidative stress (76). SphK1 also mitigates palmitate-induced ER stress-associated apoptosis in hepatocytes (65). In contrast, as a proinflammatory and profibrogenic factor, SphK1 promotes NAFLD progression to non-alcoholic steatohepatitis (NASH). The hepatic SphK1 mRNA level is profoundly increased in subjects with advanced fibrotic livers (77). Ablation of *Sphk1* ameliorates CCl_4_ or bile duct ligation (BDL)-induced liver fibrosis in mice, at least in part, by suppressing the activation and migration of hepatic stellate cells and Kupffer cells (78). SphK1 promotes protein kinase C-δ activation, triggers NFκB signaling and promotes proinflammatory cytokine production, contributing to D-GaIN and lipopolysaccharide-induced acute liver failure (79). The role of SphK1 in steatosis is context-dependent. Overexpression of SphK1 by adeno-associated virus (AAV) in the liver reduces the level of hepatic triglyceride in mice on a low fat, but not high fat, diet (80). In contrast, knockout of SphK1 suppresses hepatosteatosis in mice on a high-fat diet (HFD) (81).

SphK1 was hypothesized to reinforce hepatic insulin sensitivity as it converts ceramides to S1P. On the one hand, ceramides, in particular, C16 ceramide, impairs hepatic insulin signaling (82, 83). In normal livers, the predominant form of ceramides is C24 ceramide that is believed to be beneficial to insulin sensitivity (84). However, feeding mice with a diet rich in saturated fat can selectively increase the C16 ceramide level in the liver, which represents an explanation of the hepatic insulin resistance in diet-induced obese mice (82). On the other hand, S1P activates PI3K/Akt pathway *via* its receptors (85, 86). In support of this notion, overexpression of SphK1 significantly enhances hepatic insulin signaling and improves glucose tolerance in KK/Ay diabetic mice (87). Acid sphingomyelinase-induced Akt phosphorylation is abrogated in livers of *Sphk1^-/-^* C57BL/6 mice (88). However, other studies hold a conclusion against this notion. Depletion of *Sphk1* does not alter insulin sensitivity in HFD-fed C57BL/6 mice (65). Liver-specific overexpression of SphK1 has no impact on insulin sensitivity and glucose tolerance in high-fat, high sugar diet-fed C57BL/6 mice (80). The phospho-Akt levels are indistinguishable in the livers of wild-type and *Sphk1^-/-^* mice upon insulin challenge (89). The insulin response is unchanged in SphK1-knockdown hepatocytes as compared with control cells (7). Furthermore, S1P impairs hepatic insulin signaling *via* binding to S1P receptor subtype 2 in HFD-fed New Zealand Obese mice (90). Therefore, to characterize the hepatocyte-autonomous role of SphK1 in hepatic insulin signaling, the use a tissue-specific knockout mouse model is recommended. Recently, the liver-specific *Sphk1^-/-^* C57BL/6 mice have been generated (91). This mouse strain displays no prominent physiological and pathological abnormalities under a basal condition (91). However, it is still intriguing to know if the liver-specific knockout of *Sphk1* affects the metabolic vulnerability of mice upon dietary or other pathologic insults.

In general, research on SphK2 is not as comprehensive as SphK1 due to its complex subcellular localization. However, the metabolic role of SphK2 in the liver has been recently addressed. SphK2 appears a metabolically protective factor in the liver. SphK2 protects mice from both alcoholic and non-alcoholic fatty liver disease (92, 93). Ablation of *Sphk2* results in an elevated level of hepatic lipid accumulation and proinflammatory cytokine production, predisposing to liver injury in C57BL/6 mice exposed to 60-day chronic alcohol feeding (92). *Sphk2^-/-^* also promotes the establishment of hepatosteatosis in mice after 2-week HFD feeding (93).

SphK2-mediated regulation of hepatic insulin signaling is likely related to its subcellular localization. Pharmacological inhibition or siRNA-mediated knockdown of SphK2 mitigates insulin-repressed gluconeogenic gene expression, leading to increased hepatic glucose production in C57BL/6 mice treated with interleukine-6 (94). This effect is reported to attribute to the dephosphorylation and deacetylation of STAT3 (94). Overexpression of SphK2 in the liver reinforces mitochondrial fatty acid β-oxidation and effectively ameliorates hepatic steatosis, glucose intolerance and insulin resistance in C57BL/6 mice on an HFD (95). We have recently generated liver-specific *Sphk2^-/-^* (SphK2-LKO) mice by crossbreeding floxed *Sphk2* mice with Alb-*Cre* strain, providing a reliable model to further characterize the role of SphK2 in the liver (7). SphK2-LKO mice exhibit impaired glucose homeostasis and insulin responsiveness on both normal diet and HFD conditions. SphK2-LKO upregulates gluconeogenic genes and downregulates glucose disposal genes, leading to an increase in hepatic glucose production. Inhibition of SphK2 by the selective inhibitors or shRNA-mediated SphK2 knockdown in hepatocytes significantly suppresses insulin-induced activation of the phosphoinositide 3-kinase (PI3K)/Akt signaling pathway (7). Interestingly, treatment of SphK2-deficient hepatocytes with S1P fails to revert insulin signaling. By contrast, myriocin, an inhibitor that blocks the biosynthesis of all sphingolipids, including S1P, can restore insulin-induced Akt phosphorylation, indicating that S1P is unrelated to SphK2-mediated regulation of hepatic insulin signaling (7). Furthermore, inhibition of ceramide synthesis by fumonisin b1 has no impact on hepatic insulin sensitivity, whereas blocking ceramide degradation *via* pharmacological inhibition or knockdown of acid ceramidase restores insulin sensitivity in SphK2-deficient hepatocytes (7). This finding supports the notion that hepatic ceramide levels are often unrelated to hepatic insulin sensitivity in humans and rodents [as reviewed in (75)]. Notably, we found that hepatic levels of sphingosine are critically associated with insulin sensitivity both *in vitro* and *in vivo* (7). Moreover, treatment of hepatocytes with sphingosine significantly inhibits the insulin-induced PI3K activity and Akt phosphorylation (7). However, how sphingosine inhibits PI3K remains unknown. It has been demonstrated that sphingosine can both physically and functionally interact with the protein 14-3-3ζ (96), which, in turn, promotes plasma membrane recruitment and activation of PI3K (97, 98) To what extent this pathway contributes to the regulation of hepatic insulin signaling warrants further investigations.

## Sphingosine Kinase and Insulin Resistance in Skeletal Muscle

The major contributions of skeletal muscle in maintaining euglycemia are glucose uptake and disposal (99). In both postprandial state and the hyperinsulinemic-euglycemic clamp experiments, skeletal muscle uptakes approximately 80% of blood glucose (100, 101). The insulin signaling pathway in skeletal muscle is also centered with PI3K/Akt, specifically promoting Akt-driven glucose uptake, utilization and storage *via* the regulation of glucose transporter type 4 (GLUT4) (102). Lipid overload is also a chief cause of insulin resistance in myocytes. HFD feeding or lipid infusion causes diacylglycerol accumulation, which activates protein kinase C θ, negatively regulating insulin signaling in skeletal muscle (103–105).

HFD feeding of mice or treatment of C2C12 myoblasts with palmitate results in a dramatic increase in myocyte SphK1 mRNA expression (106). Notably, in these lipid-induced insulin-resistant myocytes or muscle tissues, levels of sphingolipids, including S1P, are all increased (106). Levels of ceramide, sphingosine and S1P are also profoundly increased in palmitate-treated L6 myotubes (72). By far, the majority of studies support a metabolically protective role of SphK1 in muscle insulin resistance. For instance, adenoviral overexpression of SphK1 enhances, whereas siRNA-mediated knockdown of SphK1 suppresses insulin-mediated glucose uptake in murine C2C12 myoblasts (87). In line with this, transgenic overexpression of SphK1 that promotes intramuscular ceramide conversion into S1P significantly improves muscle and whole-body insulin resistance in mice on an HFD for 6 weeks (70). In addition, S1P can trans-activate myocyte insulin signaling in the absence of insulin stimulation and thus enhance glucose uptake (107). Inhibition of sphingolipid synthesis by myriocin restores, whereas inhibition of SphKs by non-selective inhibitor SKI-II further suppresses, the impaired insulin-mediated glucose uptake in L6 myotubes exposed to palmitate (72). Interestingly, the level of sphingosine, but not ceramide or S1P, is always correlated to insulin’s actions in these experimental settings, suggesting an important role for sphingosine (72). However, Ross et al. reported that SphK1/S1P promotes muscle insulin resistance in HFD-fed C57BL/6 mice through S1P3 receptor-dependent elevation of interleukin-6 production by skeletal muscle (108). Thus, the role of SphK1/S1P in muscle insulin resistance appears contradictory in the literature, which needs further clarification, especially by using myocyte-specific *Sphk1^-/-^* mice.

There is no direct experimental evidence depicting the role of SphK2 in muscle insulin resistance. Transiently overexpression of SphK2 in skeletal muscle using *in vivo* electroporation has no impact on sphingomyelin and ceramide levels (70). However, the study did not determine the levels of sphingosine and S1P or insulin sensitivity (70). The same research group has later found that oral administration of FTY720 for 6 weeks abrogates lipid accumulation and improves glucose uptake in the skeletal muscle of C57BL/6 mice on an HFD (109). FTY720 elicits two possible working mechanisms: a) It can be phosphorylated into FTY-720-P, primarily by SphK2, and act on S1P receptors as an S1P mimetic (110); b) It functions as an inhibitor of SphK (both SphK1 and SphK2) and ceramide synthases (111–113). Nevertheless, how FTY720 improves muscle insulin resistance and whether SphK2 is involved in this regulation remain elusive.

## Sphingosine Kinase and Insulin Resistance in White Adipose Tissue

White adipose tissue is the main reservoir of fat storage in our body. In each adipocyte, fat is usually stored in the form of triglyceride in a unilocular lipid droplet (6). When needed, white adipose tissue expands its fat storage capacity by increasing either the number (hyperplasia) or size (hypertrophy) of adipocytes, leading to obesity (6). These two types of adipose tissue expansion result in distinct metabolic outcomes. Insulin resistance is often associated with hypertrophic obesity but not adipocyte hyperplasia (114, 115). In recent years, the expandability of white adipose tissue is emerging as a widely accepted concept to explain to non-obese/insulin-resistant and obese/insulin-sensitive populations (6). When excess fat overwhelms the storage capacity of white adipose tissue, ectopic lipid accumulation will occur in the liver, skeletal muscle and heart, resulting in metabolic disorders (6). For example, severe insulin resistance is seen in patients with congenital generalized lipodystrophy, a condition in which white adipose tissue is near completely lost (116). White adipose tissue also functions as an endocrine organ regulating glucose homeostasis *via* secretion of adipokines, e.g., adiponectin, resistin and leptin that improve metabolic profile (117, 118), or the cytokines that impair adipose tissue and whole-body insulin responsiveness, linking obesity to T2D (119).

Recently, two important studies on the role of SphK1 in adipose insulin resistance have been reported by Cowart laboratory (89, 120). They found that SphK1 expression is upregulated in both palmitate-treated white adipocytes *in vitro* and white adipose tissue in mice on an HFD *in vivo* (89). After 16 weeks of HFD feeding, *Sphk1^-/-^* mice develop a comparable body weight as wild-type counterparts, but they exhibit prominent adipocyte hyperplasia and upregulation of adipogenic transcription factors (89). As a result, *Sphk1^-/-^* significantly improves whole-body metabolic abnormalities and, particularly, rescues Akt signaling in white adipose tissue of HFD-fed mice (89). This is attributed to the alleviation of a proinflammatory phenotype in white adipose tissue (89). In support of this, SphK1 is found to promote adipose tissue macrophage survival and interleukin-6 production (121, 122). To further elucidate the adipose tissue-specific role of SphK1, Cowart laboratory has generated SphK1^fatKO^ mice on a C57BL/6 background by crossbreeding floxed SphK1 mice with Adipoq-*Cre* strain (120). In marked contrast to the anti-diabetic effects of global depletion of *Sphk1*, SphK1^fatKO^ unexpectedly causes severely impaired glucose tolerance and significantly elevated fasting glucose in diet-induced obese mice (120). Mechanistically, SphK1^fatKO^ suppresses adipocyte lipolysis, leading to hypertrophic obesity (120). This work clearly indicates that SphK1 is essential in adipose tissue to prevent metabolic abnormalities. It also suggests that the anti-diabetic benefits seen in global *Sphk1^-/-^* mice might derive be related to the proinflammatory effects of SphK1in other cell types (89, 120). By comparing these two studies, we are more aware of the necessity of using tissue-specific genetically modified mice in SphK studies.

Opposing to *Sphk1^-/-^* that increases adiposity, the global ablation of *Sphk2* progressively decreases fat mass in standard chow diet-fed mice along with aging (123). In aged mice at 50–52 weeks old, depletion of *Sphk2* profoundly elevates glucose tolerance in both male and female mice (123). In males but not female mice, *Sphk2^-/-^* improves insulin sensitivity to a much lesser extent (123). Dramatically reduced white adipose tissue mass can be partially attributable to the increased adipocyte lipolysis and energy expenditure (123). Taking the lesson from *Sphk1^-/-^* models as mentioned above, the adipose-specific actions of SphK2 warrant further investigations using adipose-specific knockout mice.

## Sphingosine Kinases and Pancreatic β-Cell Failure

Pancreatic β-cells are the only source of insulin production. Insulin secretion is primarily stimulated by glucose, which, in turn, lowers the glucose level in the circulation (124). Pancreatic β-cell failure, including both dysfunction and cell death, is the fundamental cause of hyperglycemia in diabetes. In prediabetic states, although insulin resistance exists, the blood glucose level is just subtly increased, which is attributed to a compensated hypersecretion of insulin by β-cells (125, 126). When β-cell compensation fails due to either dysfunction or cell death, hyperglycemia takes place as a key feature of the onset of diabetes (125, 126). Aberrant lipid accumulation is a chief factor of inducing β-cell dysfunction and death, designated as β-cell lipotoxicity, which is often seen in obese and prediabetic subjects (125, 126).

SphK1 and SphK2 are both suggested to be implicated in the regulation of β-cell’s insulin secretory function. In rat INS-1β-cell line and primary islets, SphK2 is the predominant isoform of SphK, whereas SphK1 is hardly detected (34). In contrast, SphK1 level is induced by cytokine challenge, whereas SphK2 expression is unchanged (34). Inhibition of SphK1 and SphK2 by the inhibitor SKI impairs glucose-stimulated insulin secretion (GSIS) in both MIN6 β-cell line and C57BL/6 mice (127). In line with this, blocking S1P dephosphorylation into sphingosine by knockdown of S1P phosphatase-1 enhances insulin production in MIN6 cells (127). These data indicate that the sphingolipid rheostat is a determinant of GSIS in β-cells. However, it is controversial in terms of which isoform of SphK is primarily responsible for this regulation. Hasan et al. reported that SphK1 knockdown suppresses GSIS in INS-1 cells (128). On the contrary, Cantrell et al. found that knockdown of SphK2, but not SphK1, abolishes GSIS in MIN6 cells (127). This discrepancy is yet to be resolved.

As it has been documented in many other types of cells, SphK1 and SphK2 are both implicated in the regulation of β-cell viability. Overexpression of SphK1 inhibits palmitate-induced lipotoxicity in INS-1 cells exposed to high glucose (30 mM) (129). This effect is independent of S1P receptors, as the inhibitors or antagonists of these receptors cannot compromise SphK1-mediated protection (129). According to the lipidomic analyses, overexpression of SphK1 mainly reduces the level of C16 ceramide in palmitate-treated INS-1 cells under low glucose (5 mM) condition, but C18, C24 and C26 ceramides in high glucose condition (129). It has been shown that ER-to-Golgi trafficking is essential for ceramide-induced lipotoxicity in β-cells (130). Interestingly, overexpression of SphK1 prevents palmitate-mediated impairment of ER-to-Golgi protein transport (129). Our laboratory has recently demonstrated a protective role of SphK1 in β-cell lipotoxicity *in vivo* (131). We found that ablation of *Sphk1* promotes the development of impaired fasting glucose and impaired glucose tolerance in C57BL/6 mice on an HFD for 24 weeks. In marked contrast to hyperinsulinemia found in wild-type littermates, *Sphk1* ^-/-^ mice exhibit significantly reduced levels of plasma insulin. Under a similar degree of whole-body insulin resistance, *Sphk1* ^-/-^ potentiates lipid-induced apoptosis of pancreatic β-cells, leading to the diabetic phenotype. The antiapoptotic effects of SphK1 are reproducible in palmitate-treated isolated islets *ex vivo* (130). In marked contrast to the protective role of SphK1, we have recently identified SphK2 as an endogenous proapoptotic factor in β-cells, profoundly promoting lipotoxicity (40). Ablation of *Sphk2* partially reserves β-cell mass and thus improves diabetic phenotype in an animal model of T2D (treated with a combination of HFD and streptozotocin) (40). In addition, knockdown of SphK2 by siRNA or inhibition of SphK2 by ABC294640 abrogates palmitate-induced apoptosis in INS-1 cells (40). Mechanistically, palmitate stimulates nuclear export of SphK2 to the cytoplasm, where SphK2 mediates mitochondria-dependent apoptosis signaling *via* its BH3 domain (40). Taken together, our findings that SphK1 protects but SphK2 promotes β-cell lipotoxicity suggest a new strategy by balancing between the signaling of SphK1 and SphK2 in β-cells for the prevention and treatment of diabetes.

## Conclusion

**Figure 2:**
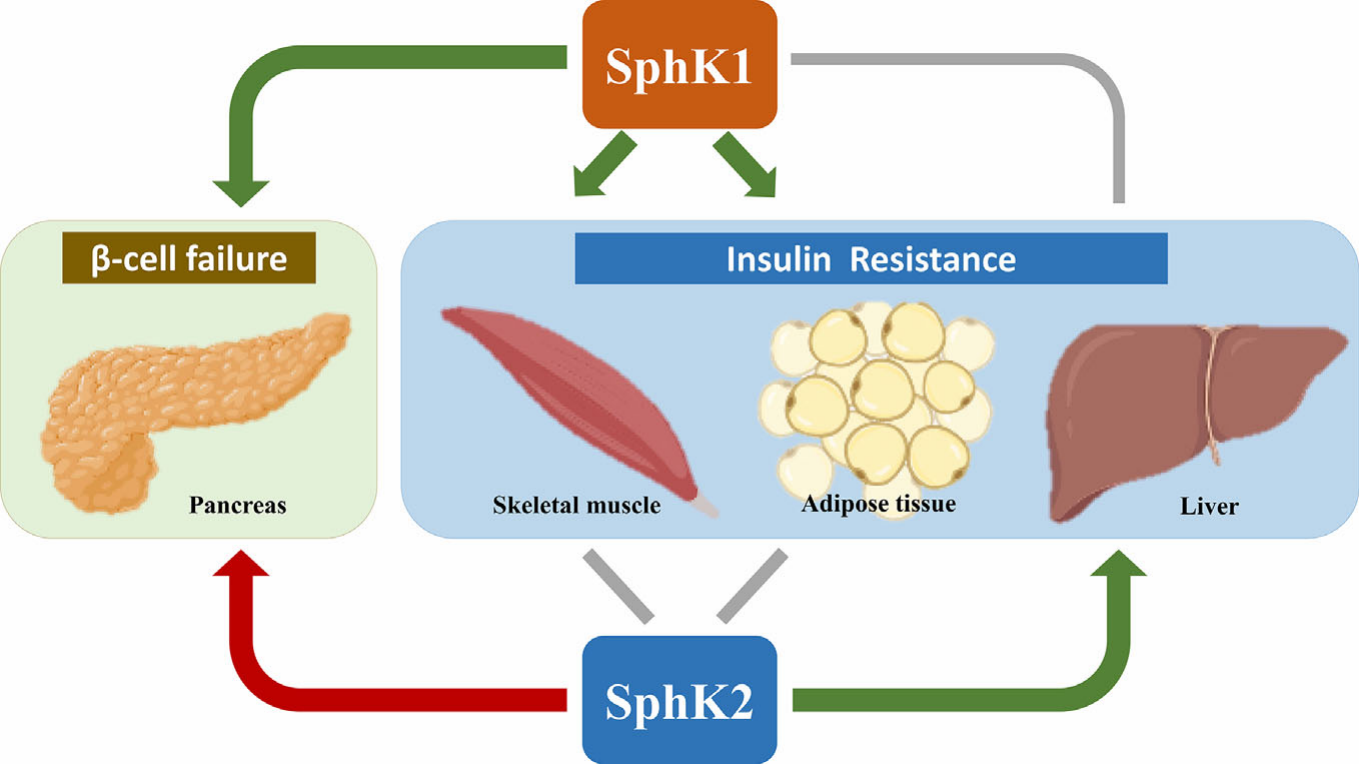
Roles of SphK1 and SphK2 in T2D. Most of the literature supports an anti-diabetic role (denoted by green arrows) of SphK1 in skeletal muscle, adipose tissue and pancreas as well as SphK2 in the liver. In contrast, SphK2 is primarily a pro-diabetic factor (denoted by the red arrow) for its proapoptotic effects in pancreatic β-cells. The roles of SphK1 in the liver and SphK2 in skeletal muscle and adipose tissue are controversial (denoted by grey lines). Created in BioRender.com.

Over the last decade, there have been extensive efforts to explore the potential roles of SphK1 and SphK2 in T2D (**Figure 2**). In hepatic insulin resistance, SphK2 has been illustrated as a metabolically protective factor, whereas the effects of SphK1 are controversial. In muscle insulin resistance, the role of SphK1 is still under debate, while little is known about SphK2. In white adipose tissue, SphK1 prevents obesity-associated diabetes, whereas the adipose-specific role of SphK2 remains elusive. Both SphK1 and SphK2 have been found essential for GSIS in pancreatic β-cells; however, which of the two isoforms takes in lead warrants further clarification. Remarkably, SphK1 and SphK2 exert opposite effects in protecting β-cells from lipotoxicity.

It should be taken into consideration in further studies that the complicated roles for the two isoforms of SphK in T2D are related to multiple layers of complexities as follows: i) SphK1 versus SphK2. The two isoenzymes sometimes exhibit the same effects, as they share common catalytic actions in the conversion of ceramide/sphingosine into S1P, while they sometimes lead to discrepant pathophysiological outcomes due to their distinct tissue distribution, subcellular localization and molecular partners. ii) Prosurvival versus proinflammatory role of SphK1. SphK1 simultaneously possess both prosurvival and proinflammatory properties. However, its prosurvival effects are often associated with an anti-diabetic outcome, whereas proinflammatory signaling could promote the development of diabetes. This controversy can be seen in research on hepatic and muscle insulin resistance. In addition, the opposite metabolic profiles from global and adipose-specific *Sphk1* ^-/-^ mice draw our attention to that the proinflammatory actions of SphK1 might derive from the immune cells in the insulin target tissue, but not parenchymal cells. iii) S1P receptors. S1P receptor subtypes 1-5 have been implicated in a variety of cell signaling pathways, which can lead to different and even opposite biological functions. For any S1P-related regulation, SphK and S1P receptors should be co-investigated, particularly *in vivo*. iv) Sphingolipid metabolites. Increasing evidence has shown that not only ceramides and S1P, but also many other sphingolipid metabolites, such as sphingosine, sphingomyelin and ganglioside, function as critical regulators of insulin actions in different experimental settings. This notion has brought new possibilities for a better explanation of the role of SphK and sphingolipids in the pathogenesis of diabetes.

In conclusion, since identified in the late 1990s, SphK1 and SphK2 have been extensively studied in a wide variety of biomedical fields. Their potential roles in T2D have just been explored recently and remain to be further characterized. Tissue-specific knockout mice are powerful tools to dissect the cell-autonomous effects of SphK. So far, only hepatocyte-specific *Sphk2* ^-/-^ and adipocyte-specific *Sphk1* ^-/-^ have been applied in diabetes research. In addition, with the development of sphingolipidomic analyses, a more precise and comprehensive measurement of sphingolipid composition in different pathophysiological contexts should be performed. Furthermore, the knowledge related to genetic and epigenetic regulation of SphK1 and SphK2in T2D remains largely unknown. Nevertheless, a comprehensive understanding of the role of SphK and the associated sphingolipids in T2D will help to identify druggable targets for prevention and treatment of the disease in the future.

## Author Contributions

PX and YQ conceived and wrote the manuscript. WW, ZS, GA, and XL provided technical assistance. All authors contributed to the article and approved the submitted version.

## Funding

This study was supported by National Natural Science Foundation of China (NSFC)–National Health and Medical Research Council, Australia (NHMRC) Joint Research Grants 81561128014 (to PX) and APP1113527 (to Mathew A Vadas), NSFC Grants 81370937 and 81870559 (to PX), NHMRC Project Grant APP1162545 (to YQ) and Fudan Distinguished Professorship (to PX).

## Conflict of Interest

The authors declare that the research was conducted in the absence of any commercial or financial relationships that could be construed as a potential conflict of interest.
